# Association of the blood urea nitrogen to serum albumin ratio and 28-day all-cause mortality in patients with cardiac arrest: a retrospective cohort study using the MIMIC-IV database

**DOI:** 10.3389/fcvm.2025.1609059

**Published:** 2025-06-06

**Authors:** Gaosheng Zhou, Yayuan Tan, Xueli Li, Yixun Wang, Dingdeng Wang, Min Liu

**Affiliations:** ^1^ The First College of Clinical Medical Science, China Three Gorges University, Yichang, China; ^2^ Department of Critical Care Medicine, Yichang Central People’s Hospital, Yichang, China; ^3^ Yichang Sepsis Clinical Research Center, Yichang, Hubei, China

**Keywords:** cardiac arrest, blood urea nitrogen, serum albumin, mortality, prognosis

## Abstract

**Background:**

The blood urea nitrogen to serum albumin ratio (BAR) has been identified as a novel indicator of both inflammatory and nutritional status, exhibiting a correlation with adverse cardiovascular outcomes.

**Objective:**

To explore the association between the BAR and 28-day all-cause mortality in cardiac arrest patients who achieved return of spontaneous circulation (ROSC) and were admitted to the intensive care unit (ICU).

**Methods:**

Data for patients with cardiac arrest were obtained from the Medical Information Mart for Intensive Care IV database. The outcome was 28-day all-cause mortality. Multivariable-adjusted Cox regression analysis, curve fitting, and threshold effects analysis were used to assess the relationship between the BAR and 28-day all-cause mortality in patients with cardiac arrest in the intensive care unit.

**Result:**

A total of 793 patients were included and divided into tertiles based on the BAR (Q1, Q2, Q3); 8-day all-cause mortality rates were 37.5%, 53.4%, and 63.8%, respectively (*P* < 0.001). A higher BAR at initial admission was significantly associated with an increased 28-day all-cause mortality risk. Results from the adjusted Models 2, 3, 4, and 5 were consistent with those of Model 1. Subgroup analysis revealed no interactions in age, sex, renal disease, liver disease, vasoactive drug use, ventilation, race, aids, malignant cancer, diabetes, peptic ulcer disease, rheumatic disease, chronic pulmonary disease, cerebrovascular disease, peripheral vascular disease, congestive heart failure and myocardial infarct between the BAR and 28-day all-cause mortality. Restricted cubic spline analysis revealed a nonlinear association between the BAR and 28-day all-cause mortality (*P* = 0.003). With BAR ≤ 17.981, each 1-unit increase in the BAR was associated with a 5.7% higher risk of death [95% CI (1.012–1.105), *P* < 0.05].

**Conclusion:**

This study identified a non-linear relationship between the BAR and 28-day all-cause mortality in patients with cardiac arrest.

## 1 Introduction

Cardiac arrest is a major public health burden, as a leading cause of death and disability, with approximately 350,000 cardiac arrests occurring outside the hospital annually in the United States (1). With advances in treatment and supportive care, survival rates remain low (typically less than 10%), highlighting the urgent need for better prevention and emergency response strategies (2).

The sudden loss of effective circulation and respiratory function can lead to an immediate life-threatening situation, such as non-occlusive mesenteric ischemia and other circulatory hypoperfusion-related complications (3, 4), and timely intervention is crucial for improving patient outcomes. Despite advancements in emergency medical services and post-resuscitation care (5), the 28-day all-cause mortality rate among patients with cardiac arrest remains high (6). Therefore, a comprehensive understanding of 28-day mortality and its related factors may contribute to its prevention and control, thereby improving prognosis and quality of life among affected patients.

The blood urea nitrogen to serum albumin ratio (BAR) is a crucial blood measurement parameter that serves as a key indicator of physiological health (7). The BAR has an important role in assessing the overall well-being and functioning of the human body. This measure has shown superior predictive value for prognosis in various diseases, including sepsis, pneumonia, chronic obstructive pulmonary disease, and acute ischemic stroke (8–11). However, there is little scientific research on the correlation between the BAR and outcomes in patients with cardiac arrest. This study was undertaken to explore the prognostic value of the BAR for 28-day mortality among patients who experience cardiac arrest.

## 2 Materials and methods

### 2.1 Data source

This was a retrospective study based on data retrieved from the Medical Information Mart for Intensive Care IV (MIMIC-IV) v3.0 database, which includes critically ill patients admitted to an intensive care unit (ICU) or the emergency department of the Beth Israel Deaconess Medical Center (Boston, MA, USA) between 2008 and 2019. One of the authors passed the Collaborative Institutional Training Initiative program course required to access the database and obtained study approval from the Institutional Review Board of Beth Israel Deaconess Medical Center.

### 2.2 Study population

This study included adult patients admitted to the ICU after achieving return of spontaneous circulation (ROSC) following cardiac arrest (12). We included patients in the MIMIC-IV v2.2 database who were aged ≥18 years, admitted to ICU for the first time, and diagnosed with cardiac arrest at hospital admission according to diagnostic codes of the International Classification of Diseases Ninth Revision and Tenth Revision. The exclusion criteria were as follows: (I) not the first ICU admission; (II) age <18 years; (III) pregnancy or in the postpartum period; (IV) missing data for blood urea nitrogen (BUN) and serum albumin (ALB); (V) admission >24 h earlier; (VI) multiple admissions.

### 2.3 Extraction of variables

We used PostgreSQL version 10.13 to extract data from MIMIC-IV v2.2 regarding patients' baseline characteristics, including age, sex, comorbidities, and laboratory test results.

We extracted information from the first document containing data for vital signs and laboratory test data for patients with cardiac arrest who were admitted to the hospital. Demographic data included age, sex, and race. Vital signs data included body temperature (T), mean blood pressure (MBP), heart rate (HRT), respiratory rate (RR), and pulse oximetry-derived oxygen saturation (SPO_2_). Comorbid conditions included the Charlson Comorbidity Index (CCI), myocardial infarct, congestive heart failure, peripheral vascular disease, cerebrovascular disease, dementia, chronic pulmonary disease, rheumatic disease, peptic ulcer disease, diabetes, paraplegia, renal disease, malignant cancer, liver disease, metastatic solid tumor, and AIDS. Laboratory test results within the first 24 h were extracted from the time of admission to the ICU and included white blood cell count, hemoglobin (Hb), platelet count (PLT), alkaline phosphatase (ALP), alanine aminotransferase, aspartate aminotransferase, total bilirubin (TBIL), creatinine (Cr), glucose, sodium, potassium (K^+^), chloride (CI^−^), calcium, lactate (Lac), pH, partial pressure of carbon dioxide, partial pressure of oxygen/fraction of inspired oxygen, base excess (BE), prothrombin time (PT), and partial thromboplastin time (PTT). We also recorded scoring systems applied in the ICU including the Sequential Organ Failure Assessment (SOFA) score and Simplified Acute Physiology Score II (SAPS II). Therapy included the use of vasoactive drugs, mechanical ventilation, and renal replacement therapy (RRT) during hospitalization. The length of stay in the ICU and 28-day all-cause mortality were also recorded.

### 2.4 Statistical analysis

To minimize bias from missing data imputation, variables with ≥25% missing values were excluded; the remaining missing values were handled using multiple imputation (13)*.* The number of imputations was five. Continuous variables were imputed using linear regression models through an iterative computational process to ensure stability. The final results were obtained by pooling estimates across multiply imputed datasets, providing unbiased statistical inference under the missing-at-random (MAR) assumption.

Continuous variables that exhibited a normal distribution are expressed as mean ± standard deviation; otherwise, these are expressed as median (interquartile range). Categorical variables are reported as frequency and percentage. To explore the association between different BAR levels and 28-day all-cause mortality, enrolled patients were categorized into three groups according to the tertile distribution of BAR values within the study cohort (with the 33rd and 66th percentiles used as cutoff points).

We used the *t*-test for continuous variables with a normal distribution, the Mann–Whitney U test for continuous variables with a skewed distribution, and the chi-squared test for categorical variables to analyze differences between the two groups.

Univariable and multivariable Cox regression analyses were performed to examine the relevance between an increased serum BAR and risk of 28-day all-cause mortality in the ICU. Restricted cubic splines analysis was applied to detect the association between the BAR and risk of 28-day all-cause mortality. Kaplan–Meier analysis was conducted to compare the cumulative survival rate between the high and low BAR groups using the log-rank test. The survival rates among different groups were then compared using log-rank tests.

Variables that were considered potential confounders based on the existing literature and clinical judgment were included. Five regression models were built, and the results are presented as hazard ratio (HR) with 95% confidence interval (CI). Model 1 was unadjusted. Model 2 adjusted for age and sex. Model 3 further adjusted for the Charlson Comorbidity Index, myocardial infarct, congestive heart failure, peripheral vascular disease, cerebrovascular disease, dementia, chronic pulmonary disease, rheumatic disease, peptic ulcer disease, diabetes, paraplegia, renal disease, malignant cancer, liver disease, metastatic solid tumor, and AIDS, based on Model 2. Model 4 further adjusted for white blood cell count, Hb, platelet count, ALP, alanine aminotransferase, aspartate aminotransferase, TBIL, Cr, glucose, sodium, K^+^, CI^−^, calcium, Lac, pH, partial pressure of carbon dioxide, partial pressure of oxygen/fraction of inspired oxygen, BE, PT, partial thromboplastin time, vasoactive drug use, RRT, mechanical ventilation, and length of ICU stay, based on Model 3. Model 5 further adjusted for HRT, MBP, RR, T, SPO_2_, SAPS II, and SOFA, based on Model 4.

Subgroup analyses were performed according to age, sex, renal disease, liver disease, vasoactive drugs, rrt, ventilation, race, aids, malignant cancer, paraplegia, diabetes, peptic ulcer disease, rheumatic disease, chronic pulmonary disease, cerebrovascular disease, peripheral vascular disease, congestive heart failure and myocardial infarct. Data analyses were completed using Stata version 17.0 and IBM SPSS version 26 (IBM, Armonk, NY, USA). Statistical significance was defined as a two-tailed *P*-value <0.05.

## 3 Results

### 3.1 Baseline characteristics

**Figure 1:**
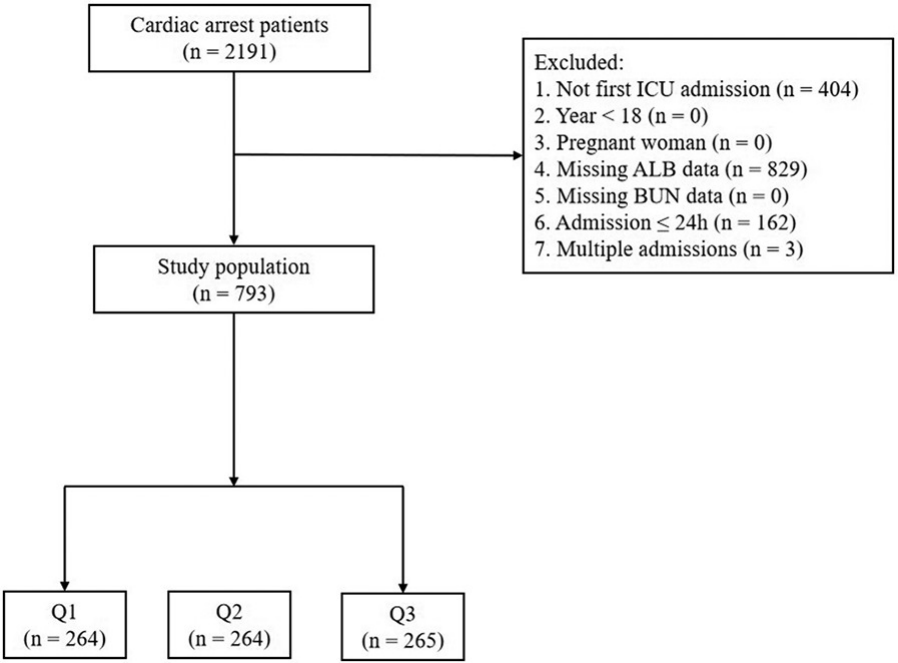
Flowchart of study participants. Patients were grouped into tertiles by BAR levels (Q1, Q2, Q3). BAR, blood urea nitrogen (BUN) to serum albumin (ALB) ratio; ICU, intensive care unit.

A total of 793 patients were ultimately enrolled in the study (Figure 1). The enrolled patients were divided into three groups according to tertiles of the BAR, the Q1, Q2, and Q3 groups. The mean age of study participants was 65.1 ± 16.9 years, 498 (62.8%) participants were men, and the 28-day all-cause mortality rate among patients with cardiac arrest was 51.6% (409/793). Patients in the group with a higher BAR(Q3) were older, predominantly men, and had higher values for SAPS II, SOFA, the Charlson Comorbidity Index, ALP, TBIL, Cr, BUN, K^+^, Lac, BE, and PT, as well as a higher prevalence of renal disease, congestive heart failure, peripheral vascular disease, diabetes, renal disease, and liver disease. Additionally, the use of vasoactive drugs and RRT was more common. With increased BAR, the values for MBP, SPO_2_, CI^−^, PO_2_/FiO_2_, Hb, and ALB showed a tendency to decrease. The 28-day all-cause mortality showed a significant incremental trend between BAR groups. The difference in mortality rates between the three groups (Q1, Q2, Q3) was statistically significant (37.5% vs. 53.4% vs. 63.8%, respectively; *P* < 0.001), suggesting that the BAR is associated with the risk of 28-day all-cause mortality (Table 1).

**Table 1:** Baseline characteristics of patients.

Variables	Total (*n* = 793)	Q1 (*n* = 264)	Q2 (*n* = 264)	Q3 (*n* = 265)	*P*
Age (years, Mea*n* ± SD)	65.1 ± 16.9	58.4 ± 17.6	67.2 ± 16.0	69.5 ± 15.2	<0.001
White	441 (55.6)	133 (50.4)	141 (53.4)	167 (63.0)	0.008
Male	498 (62.8)	153 (58.0)	166 (62.9)	179 (67.5)	0.074
HRT (/min, Mean ± SD)	84.3 ± 18.9	82.2 ± 19.6	85.3 ± 18.7	85.3 ± 18.3	0.086
MBP (mmHg, Mean ± SD)	78.6 ± 11.0	82.5 ± 11.8	78.1 ± 9.7	75.2 ± 10.3	<0.001
RR (/min, Mean ± SD)	21.1 ± 4.6	20.5 ± 4.3	21.6 ± 4.8	21.1 ± 4.5	0.029
T (°C, Mean ± SD)	36.4 ± 1.2	36.4 ± 1.1	36.3 ± 1.3	36.4 ± 1.0	0.296
SPO_2_ (%, Mean ± SD)	97.0 ± 3.1	97.3 ± 2.8	97.2 ± 2.7	96.5 ± 3.7	0.003
SAPSII (Mean ± SD)	48.9 ± 16.9	39.1 ± 14.9	50.3 ± 14.8	57.1 ± 15.8	<0.001
SOFA (Mean ± SD)	8.0 ± 3.8	5.8 ± 3.4	8.4 ± 3.4	9.8 ± 3.6	<0.001
CCI (Mean ± SD)	5.5 ± 3.1	3.8 ± 2.7	5.5 ± 2.8	7.1 ± 3.0	<0.001
Myocardial infarct, *n* (%)	230 (29.0)	62 (23.5)	86 (32.6)	82 (30.9)	0.049
Congestive heart failure, *n* (%)	339 (42.7)	83 (31.4)	115 (43.6)	141 (53.2)	<0.001
Peripheral vascular disease, *n* (%)	117 (14.8)	23 (8.7)	33 (12.5)	61 (23.0)	<0.001
Cerebrovascular disease, *n* (%)	126 (15.9)	47 (17.8)	47 (17.8)	32 (12.1)	0.115
Dementia, *n* (%)	26 (3.3)	6 (2.3)	10 (3.8)	10 (3.8)	0.532
Chronic pulmonary disease, *n* (%)	205 (25.9)	59 (22.3)	77 (29.2)	69 (26.0)	0.201
Rheumatic disease, *n* (%)	25 (3.2)	8 (3.0)	6 (2.3)	11 (4.2)	0.461
Peptic ulcer disease, *n* (%)	23 (2.9)	4 (1.5)	6 (2.3)	13 (4.9)	0.051
Diabetes, *n* (%)	279 (35.2)	47 (17.8)	100 (37.9)	132 (49.8)	<0.001
Paraplegia, *n* (%)	29 (3.7)	13 (4.9)	10 (3.8)	6 (2.3)	0.262
Renal disease, *n* (%)	249 (31.4)	18 (6.8)	67 (25.4)	164 (61.9)	<0.001
Malignant cancer, *n* (%)	90 (11.3)	27 (10.2)	29 (11.0)	34 (12.8)	0.624
Liver disease, *n* (%)	161 (20.3)	35 (13.3)	61 (23.1)	65 (24.5)	0.002
Metastatic solid tumor, *n* (%)	36 (4.5)	11 (4.2)	9 (3.4)	16 (6.0)	0.327
AIDS, *n* (%)	4 (0.5)	2 (0.8)	0 (0)	2 (0.8)	0.568
WBC (x 10^9^/L, Mean ± SD)	17.9 ± 11.6	16.6 ± 12.9	18.2 ± 8.8	18.7 ± 12.5	0.098
Hb (g/L, Mean ± SD)	10.5 ± 2.5	11.3 ± 2.3	10.7 ± 2.7	9.3 ± 2.2	<0.001
PLT (x 10^9^/L, Mean ± SD)	174.6 ± 97.6	184.2 ± 90.8	167.7 ± 91.0	171.9 ± 109.4	0.130
ALP (U/L, Median)	96.0 (68.0, 141.0)	81.0 (58.0, 111.0)	96.5 (68.0, 139.0)	115.0 (81.0, 176.0)	<0.001
ALT (U/L, Median)	69.0 (30.0, 218.0)	68.0 (30.0, 174.0)	77.0 (31.8, 259.0)	64.0 (28.0, 274.0)	0.397
AST (U/L, Median)	116.0 (48.0, 363.0)	114.0 (48.0, 284.0)	126.0 (46.0, 399.2)	119.0 (51.0, 458.0)	0.110
TBIL (μmol/L, Median)	0.7 (0.4, 1.3)	0.6 (0.4, 1.0)	0.7 (0.5, 1.4)	0.8 (0.5, 1.8)	<0.001
ALB (mg/dl, Mean ± SD)	3.1 ± 0.7	3.5 ± 0.6	3.1 ± 0.6	2.7 ± 0.7	<0.001
Cr (μmol/L, Median)	1.6 (1.1, 2.7)	1.1 (0.8, 1.3)	1.7 (1.2, 2.3)	2.9 (2.1, 4.5)	<0.001
BUN (mg/dl, Median)	30.0 (20.0, 48.0)	17.0 (13.0, 21.0)	30.0 (26.0, 36.0)	60.0 (47.0, 76.0)	<0.001
Glu (mmol/L, Median)	158.3 (125.6, 208.2)	144.2 (119.3, 179.3)	172.6 (139.7, 224.5)	158.6 (121.2, 220.7)	<0.001
Na^+^ (mmol/L, Mean ± SD)	141.3 ± 7.0	141.9 ± 6.0	140.9 ± 5.3	141.2 ± 9.2	0.245
K^+^ (mmol/L, Mean ± SD)	5.2 ± 1.2	4.8 ± 1.0	5.2 ± 1.2	5.5 ± 1.2	<0.001
CI^−^ (mmol/L, Mean ± SD)	107.1 ± 7.4	108.5 ± 6.7	107.3 ± 7.4	105.6 ± 7.7	<0.001
Ca^2+^ (mmol/L, Mean ± SD)	1.2 ± 0.2	1.2 ± 0.2	1.2 ± 0.2	1.2 ± 0.2	0.647
Lac (mmol/L, Median)	3.8 (1.9, 6.6)	3.0 (1.7, 5.3)	4.1 (2.3, 7.1)	4.4 (2.1, 7.8)	<0.001
PH (Mean ± SD)	7.4 ± 0.1	7.4 ± 0.1	7.4 ± 0.1	7.4 ± 0.1	0.059
PCO_2_ (mmHg, Mean ± SD)	49.1 ± 16.2	49.8 ± 18.3	49.9 ± 15.5	47.5 ± 14.5	0.160
PO_2_/FiO_2_ (mmHg, Mean ± SD)	205.1 ± 124.9	233.2 ± 132.8	192.6 ± 121.8	189.6 ± 115.2	<0.001
BE (mmol/L, Median)	−7.0 (−12.0, −2.0)	−5.0 (−9.0, −1.0)	−8.0 (−12.0, −3.0)	−9.0 (−13.0, −2.0)	<0.001
PT (s, Median)	15.7 (13.4, 21.9)	14.1 (12.5, 16.8)	15.6 (13.7, 21.9)	19.1 (14.7, 26.3)	<0.001
PTT (s, Median)	42.1 (31.7, 81.3)	36.4 (30.0, 70.8)	43.2 (32.7, 90.8)	43.7 (33.4, 81.3)	0.005
Vasoactive drugs, *n* (%)	492 (62.0)	124 (47.0)	187 (70.8)	181 (68.3)	<0.001
RRT, *n* (%)	64 (8.1)	3 (1.1)	12 (4.5)	49 (18.5)	<0.001
Ventilation, *n* (%)	495 (62.4)	157 (59.5)	177 (67)	161 (60.8)	0.157
Los ICU (days, Median)	5.0 (3.0, 9.0)	5.0 (3.0, 9.0)	5.0 (3.0, 9.0)	5.0 (3.0, 10.0)	0.805
28-day all-cause mortality, *n* (%)	409 (51.6)	99 (37.5)	141 (53.4)	169 (63.8)	<0.001

Patients were grouped into tertiles by BAR levels (Q1, Q2, Q3).

Variables are expressed as mean ± standard deviation (SD), median (interquartile range, IQR), or *n* (%).

BAR, blood urea nitrogen to serum albumin; HRT, heart rate; MBP, mean blood pressure; RR, respiratory rate; T, temperature; SPO_2_, oxygen saturation; SAPS II, Simplified Acute Physiology Score II; SOFA, Sequential Organ Failure Assessment; CCI, Charlson Comorbidity Index; AIDS, acquired immune deficiency syndrome; WBC, white blood cell; Hb, hemoglobin; PLT, platelet count; ALP, alkaline phosphatase; ALT, alanine aminotransferase; AST, aspartate aminotransferase; TBIL, total bilirubin; ALB, albumin; Cr, creatinine; BUN, blood urea nitrogen; Glu, glucose; Na^+^, sodium; K^+^, potassium; CI^−^, chloride; Ca^2+^, calcium; Lac, lactate; PCO_2_, partial pressure of carbon dioxide; PO_2_/FiO_2_, partial pressure of oxygen in arterial blood to fraction of inspired oxygen ratio; BE, base excess; PT, prothrombin time; PTT, partial thromboplastin time; RRT, renal replacement therapy; Ventilation, mechanical ventilation; Los ICU, length of stay in intensive care unit.

### 3.2 Univariate Cox regression analysis

When the BAR was treated as a continuous variable, for each 1-unit increase in the BAR, the risk of mortality increased by 1% (HR = 1.010, 95% CI: 1.010–1.020, *P* < 0.001). Patients were divided into two equal groups (Q1, Q2) according to BAR value; the HR (Q1 for reference) was 1.79 (95% CI: 1.470–2.180, *P* < 0.001). Moreover, when patients were divided into three groups according to BAR value (Q1, Q2, Q3), the HR (Q2 vs. Q1) was 1.6 (95% CI: 1.240–2.070, *P* < 0.001) and further increased (Q3 vs. Q1) to 2.07 (95% CI: 1.610–2.650, *P* < 0.001). This indicates that the higher the BAR, the higher the risk of 28-day all-cause mortality among patients (Table 2).

**Table 2:** Results of univariate Cox analysis.

Variables	HR (95% CI)	*P* (Wald's test)
Age	1.009 (1.002, 1.015)	0.006
Sex: male vs. female	0.880 (0.730, 1.080)	0.227
Race: ref = White		
Black	1.070 (0.780, 1.470)	0.683
Other	1.500 (1.220, 1.850)	<0.001
HRT	1.010 (1.010, 1.020)	<0.001
MBP	0.994 (0.985, 1.004)	0.238
RR	1.070 (1.040, 1.090)	<0.001
T	0.780 (0.720, 0.830)	<0.001
SPO_2_	0.920 (0.890, 0.950)	<0.001
SAPSII	1.030 (1.020, 1.030)	<0.001
SOFA	1.070 (1.040, 1.100)	<0.001
CCI	1.040 (1.000, 1.070)	0.035
Myocardial infarct: yes vs. no	0.820 (0.660, 1.020)	0.082
Congestive heart failure: yes vs. no	0.710 (0.580, 0.860)	<0.001
Peripheral vascular disease: yes vs. no	1.030 (0.790, 1.350)	0.824
Cerebrovascular disease: yes vs. no	1.330 (1.030, 1.700)	0.026
Dementia: yes vs. no	1.450 (0.900, 2.320)	0.125
Chronic pulmonary disease: yes vs. no	0.960 (0.770, 1.200)	0.730
Rheumatic disease: yes vs. no	1.360 (0.810, 2.280)	0.243
Peptic ulcer disease: yes vs. no	0.690 (0.370, 1.290)	0.245
Diabetes: yes vs. no	0.950 (0.780, 1.170)	0.651
Paraplegia: yes vs. no	0.840 (0.480, 1.450)	0.526
Renal disease: yes vs. no	0.980 (0.790, 1.210)	0.840
Malignant cancer: yes vs. no	1.350 (1.020, 1.800)	0.038
Liver disease: yes vs. no	1.070 (0.840, 1.350)	0.585
Metastatic solid tumor: yes vs. no	2.330 (1.600, 3.380)	<0.001
AIDS: yes vs. no	1.020 (0.260, 4.110)	0.974
WBC	1.007 (1.001, 1.013)	0.014
Hb	0.970 (0.930, 1.010)	0.107
PLT	1.000 (0.999, 1.001)	0.490
ALP	1.002 (1.001, 1.003)	<0.001
ALT	1.000 (1.000, 1.000)	0.006
AST	1.000 (1.000, 1.000)	0.022
TBIL	1.030 (1.000, 1.070)	0.053
ALB	0.730 (0.640, 0.840)	<0.001
Cr	0.994 (0.966, 1.023)	0.688
BUN	1.007 (1.004, 1.010)	<0.001
Glu	1.000 (1.000, 1.000)	0.719
Na^+^	1.010 (1.000, 1.020)	0.007
K^+^	1.060 (0.980, 1.150)	0.143
CI^−^	1.020 (1.000, 1.030)	0.008
Ca^2+^	0.780 (0.470, 1.270)	0.316
Lac	1.090 (1.070, 1.120)	<0.001
PH	0.090 (0.030, 0.270)	<0.001
PCO_2_	0.996 (0.989, 1.002)	0.163
PO_2_/FiO_2_	0.999 (0.999, 1.000)	0.116
BE	0.970 (0.960, 0.980)	<0.001
PT	1.008 (1.004, 1.012)	<0.001
PTT	1.001 (0.998, 1.003)	0.680
Vasoactive drugs: yes vs. no	1.380 (1.130, 1.700)	0.002
RRT: yes vs. no	1.150 (0.820, 1.630)	0.417
Ventilation: yes vs. no	1.090 (0.890, 1.340)	0.388
Los ICU	0.940 (0.920, 0.960)	<0.001
BAR	1.010 (1.010, 1.020)	<0.001
BAR^a^: Q2 vs. Q1 1	1.790 (1.470, 2.180)	<0.001
BAR^b^: ref. = Q1		
Q2 vs. Q1	1.600 (1.240, 2.070)	<0.001
Q3 vs. Q1	2.070 (1.610, 2.650)	<0.001

^a^ Patients were divided into two equal groups (Q1, Q2).

^b^ Tertiles (Q1, Q2, Q3) by BAR levels.

HR, hazard ratio; CI, confidence interval; ref, reference group; BAR, blood urea nitrogen to serum albumin; HRT, heart rate; MBP, mean blood pressure; RR, respiratory rate; T, temperature; SPO_2_, oxygen saturation; SAPS II, Simplified Acute Physiology Score II; SOFA, Sequential Organ Failure Assessment; CCI, Charlson Comorbidity Index; AIDS, acquired immune deficiency syndrome; WBC, white blood cell; Hb, hemoglobin; PLT, platelet count; ALP, alkaline phosphatase; ALT, alanine aminotransferase; AST, aspartate aminotransferase; TBIL, total bilirubin; ALB, albumin; Cr, creatinine; BUN, blood urea nitrogen; Glu, glucose; Na^+^, sodium; K^+^, potassium; CI^−^, chloride; Ca^2+^, calcium; Lac, lactate; PCO_2_, partial pressure of carbon dioxide; PO_2_/FiO_2_, partial pressure of oxygen in arterial blood to fraction of inspired oxygen ratio; BE, base excess; PT, prothrombin time; PTT, partial thromboplastin time; RRT, renal replacement therapy; Ventilation, mechanical ventilation; Los ICU, length of stay in the intensive care unit.

### 3.3 Multivariate Cox regression analysis

The effect of the BAR on 28-day all-cause mortality was assessed using a Cox proportional hazard model. When the BAR was treated as a continuous variable, it was positively associated with 28-day all-cause mortality. As shown in Table 3, in the unadjusted Model 1, each unit increase in the BAR was associated with a 1% increase in the risk of 28-day all-cause mortality (HR = 1.010, 95% CI: 1.010–1.020, *P* < 0.001). In Model 2 (adjusted for age and sex), the results remained significant (*P* < 0.001). In Model 3 (Model 2, further adjusted for myocardial infarct, congestive heart failure, peripheral vascular disease, cerebrovascular disease, dementia, chronic pulmonary disease, rheumatic disease, peptic ulcer disease, diabetes, paraplegia, renal disease, malignant cancer, liver disease, metastatic solid tumor, and AIDS), the results remained significant (*P* < 0.001). In Model 4 (Model 3, further adjusted for white blood cell count, Hb, platelet count, ALP, alanine aminotransferase, aspartate aminotransferase, TBIL, Cr, glucose, sodium, K^+^, CI^−^, Lac, pH, partial pressure of carbon dioxide, partial pressure of oxygen/fraction of inspired oxygen, BE, PT, partial thromboplastin time, vasoactive drug use, RRT, mechanical ventilation, and length of ICU stay), the results remained significant (*P* < 0.001). In Model 5 (Model 4, further adjusted for HRT, MBP, RR, T, SPO_2_, SAPS II, and SOFA), the results showed significant differences (HR = 1.010, 95% CI: 1.000–1.020, *P* = 0.003).

**Table 3:** Multivariate Cox regression analysis for 28-day all-cause mortality in patients with cardiac arrest.

Variable	Modle 1		Modle 2		Modle 3		Modle 4		Modle 5	
	HR (95%CI)	*P*	HR (95%CI)	*P*	HR (95%CI)	*P*	HR (95%CI)	*P*	HR (95%CI)	*P*
BAR	1.010 (1.010–1.020)	<0.001	1.010 (1.010–1.020)	<0.001	1.020 (1.010–1.020)	<0.001	1.020 (1.010–1.030)	<0.001	1.010 (1.000–1.020)	0.003
BAR^a^										
Q1	1 (ref)		1 (ref)		1 (ref)		1 (ref)		1 (ref)	
Q2	1.790 (1.470–2.180)	<0.001	1.750 (1.430–2.150)	<0.001	2.040 (1.630–2.55)	<0.001	1.980 (1.520–2.580)	<0.001	1.690 (1.290–2.2)	<0.001
BAR^b^										
Q1	1 (ref)		1 (ref)		1 (ref)		1 (ref)		1 (ref)	
Q2	1.600 (1.240–2.070)	<0.001	1.550 (1.190–2.020)	0.001	1.730 (1.320–2.270)	<0.001	1.650 (1.240–2.210)	0.001	1.280 (0.950–1.730)	0.100
Q3	2.070 (1.610–2.650)	<0.001	2.020 (1.560–2.610)	<0.001	2.530 (1.900–3.380)	<0.001	2.700 (1.880–3.880)	<0.001	1.910 (1.310–2.770)	0.001
Trend test	<0.001		<0.001		<0.001		<0.001		0.001	

^a^ Patients were divided into two equal groups (Q1, Q2).

^b^ Tertiles (Q1, Q2, Q3) by BAR levels.

Model 1: adjusted for none.

Model 2: adjusted for Age and Sex.

Model 3: Model 2 + CCI, Myocardial infarct, Congestive heart failure, Peripheral vascular disease, Cerebrovascular disease, Dementia, Chronic pulmonary disease, Rheumatic disease, Peptic ulcer disease, Diabetes, Paraplegia, Renal disease, Malignant cancer, Liver disease, Metastatic solid tumor, and AIDS.

Model 4: Model 3 + WBC, Hb, PLT, ALP, ALT, AST, TBIL, Cr, Glu, Na^+^, K^+^, CI^−^, Ca^2+^, Lac, PH, PCO_2_, PO_2_/FiO_2_, BE, PT, PTT, Vasoactive drugs, RRT, Ventilation and Los ICU.

Model 5: Model 4 + HRT, MBP, RR, T, SPO_2_, SAPSII and SOFA.

HR, hazard ratio; CI, confidence interval; ref reference group; BAR, blood urea nitrogen to serum albumin.

Patients were divided into equal groups based on BAR values (Q1, Q2), where the Q1 group was used as the reference group; the results remained significant in the different models.

To further explore the effect of the BAR, patients were divided into three groups according to BAR values (Q1, Q2, Q3), with the Q1 group used as the reference. The results of Model 1 showed that higher initial admission in patients was associated with an increased 28-day all-cause mortality, with a gradual increase in the HR starting from Q2 (HR = 1.600, *P* < 0.001) to Q3 (HR = 2.070, *P* < 0.001). Overall, the adjusted Models 2, 3, 4, and 5 showed consistency with Model 1.

### 3.4 Subgroup and sensitivity analysis

To further investigate the impact of other risk factors on the correlation of the BAR with 28-day all-cause mortality, subgroup analyses were carried out using the following variables in stratification: age, sex, renal disease, liver disease, vasoactive drugs, rrt, ventilation, race, aids, malignant cancer, paraplegia, diabetes, peptic ulcer disease, rheumatic disease, chronic pulmonary disease, cerebrovascular disease, peripheral vascular disease, congestive heart failure and myocardial infarct. Additive interactions between the BAR and 28-day all-cause mortality were observed for rrt and paraplegia (*P* for interaction <0.05). However, significant interactions were not found for age, sex, renal disease, liver disease, vasoactive drugs, ventilation, race, aids, malignant cancer, diabetes, peptic ulcer disease, rheumatic disease, chronic pulmonary disease, cerebrovascular disease, peripheral vascular disease, congestive heart failure and myocardial infarct.

In the sensitivity analyses, among patients aged ≤65 years, each one-unit increase in the BAR was positively associated with mortality risk, although this relationship did not reach statistical significance (HR = 1.013, *p* = 0.228). In contrast, among patients aged >65 years, the association was significant (HR = 1.017, *p* < 0.05). A significant positive correlation between BAR and mortality was observed in male patients, whereas no such association was found in female patients. In patients without liver disease, AIDS, malignant cancer, Diabetes, Peptic ulcer disease, Cerebrovascular disease, Peripheral vascular disease, Myocardial infarct and in those not receiving vasoactive drugs—higher BAR correlated significantly with increased mortality (*p* < 0.05). Similarly, patients undergoing mechanical ventilation and those with congestive heart failure exhibited a significant increase in mortality risk per unit increase in BAR (*p* < 0.05); In the non-RRT group, each one-unit increase in BAR was associated with a significant rise in mortality risk, whereas in the RRT group, each one-unit increase in BAR was associated with a significant reduced in mortality risk both subgroup differences reached statistical significance (*p* < 0.05). The findings in the paraplegia and rheumatic disease subgroups were also consistent with those observed in the RRT subgroup. In the Chronic pulmonary disease subgroup analysis, BAR was significantly associated with increased mortality risk in both patients without Chronic pulmonary disease (HR = 1.014, *p* < 0.05) and those with Chronic pulmonary disease (HR = 1.034, *p* < 0.05) (Table 4).

**Table 4:** Effect of the BAR magnitude on 28-day all-cause mortality, stratified by subgroups.

Characteristics	*n*. total	*n*. event%	HR (95% CI)	*P* value	*P* (interaction)
Age					
≤65	373	164 (44)	1.013 (0.991–1.034)	0.228	0.743
>65	420	245 (58.3)	1.017 (1.003–1.030)	0.011	
Sex					
Female	295	161 (54.6)	1.000 (0.975–1.025)	0.988	0.586
Male	498	248 (49.8)	1.019 (1.007–1.031)	0.001	
Renal disease					
No	544	279 (51.3)	1.010 (0.993–1.028)	0.212	0.706
Yes	249	130 (52.2)	1.013 (0.997–1.029)	0.102	
Liver disease					
No	632	322 (50.9)	1.016 (1.004–1.027)	0.005	0.532
Yes	161	87 (54)	1 (0.969–1.030)	0.998	
Vasoactive drugs					
No	301	137 (45.5)	1.027 (1.008–1.045)	0.004	0.259
Yes	492	272 (55.3)	1.008 (0.993–1.022)	0.285	
RRT					
No	729	374 (51.3)	1.0188 (1.006–1.030)	0.001	0.022
Yes	64	35 (54.7)	0.171 (0.165–0.176)	<0.001	
Ventilation					
No	298	147 (49.3)	0.998 (0.981–1.015)	0.871	0.196
Yes	495	262 (52.9)	1.029 (1.013–1.046)	0.000	
Race					
White	441	207 (46.9)	1.012 (0.999–1.026)	0.063	0.442
Black	94	47 (50)	1.113 (1.030–1.202)	0.006	
Other	258	155 (60.1)	1.005 (0.976–1.034)	0.730	
AIDS					
No	789	407 (51.6)	1.014 (1.005–1.024)	0.003	0.711
Yes	4	2 (50)	0.928 (0–Inf)	0.999	
Malignant cancer					
No	703	354 (50.4)	1.015 (1.004–1.025)	0.004	0.659
Yes	90	55 (61.1)	0.954 (0.887–1.025)	0.200	
Paraplegia					
No	764	396 (51.8)	1.013 (1.002–1.023)	0.014	0.028
Yes	29	13 (44.8)	0.189 (0.169–0.212)	<0.001	
Diabetes					
No	514	264 (51.4)	1.018 (1.005–1.031)	0.004	0.494
Yes	279	145 (52)	1.020 (0.995–1.045)	0.108	
Peptic ulcer disease					
No	770	399 (51.8)	1.013 (1.003–1.023)	0.008	0.138
Yes	23	10 (43.5)	0.009 (0–2.14721325175122e + 38)	0.922	
Rheumatic disease					
No	768	394 (51.3)	1.015 (1.005–1.026)	0.003	0.867
Yes	25	15 (60)	0.504 (0.484–0.525)	<0.001	
Chronic pulmonary disease					
No	588	301 (51.2)	1.014 (1.003–1.026)	0.011	0.349
Yes	205	108 (52.7)	1.034 (1.004–1.065)	0.025	
Cerebrovascular disease					
No	667	332 (49.8)	1.015 (1.004–1.026)	0.006	0.07
Yes	126	77 (61.1)	1.044 (0.985–1.107)	0.140	
Peripheral vascular disease					
No	676	346 (51.2)	1.015 (1.003–1.027)	0.011	0.932
Yes	117	63 (53.8)	1.036 (1.019–1.054)	3.00 × 10^−05^	
Congestive heart failure					
No	454	253 (55.7)	1.015 (0.999–1.032)	0.066	0.128
Yes	339	156 (46)	1.017 (1.003–1.032)	0.017	
Myocardial infarct					
No	563	301 (53.5)	1.024 (1.010–1.039)	0.000	0.316
Yes	230	108 (47)	1.019 (0.994–1.044)	0.124	

Data are presented as hazard ratio (HR) and 95% confidence interval (CI).

RRT, renal replacement therapy; Ventilation, mechanical ventilation; AIDS, acquired immune deficiency syndrome.

### 3.5 Nonlinear relationship analysis

**Figure 2:**
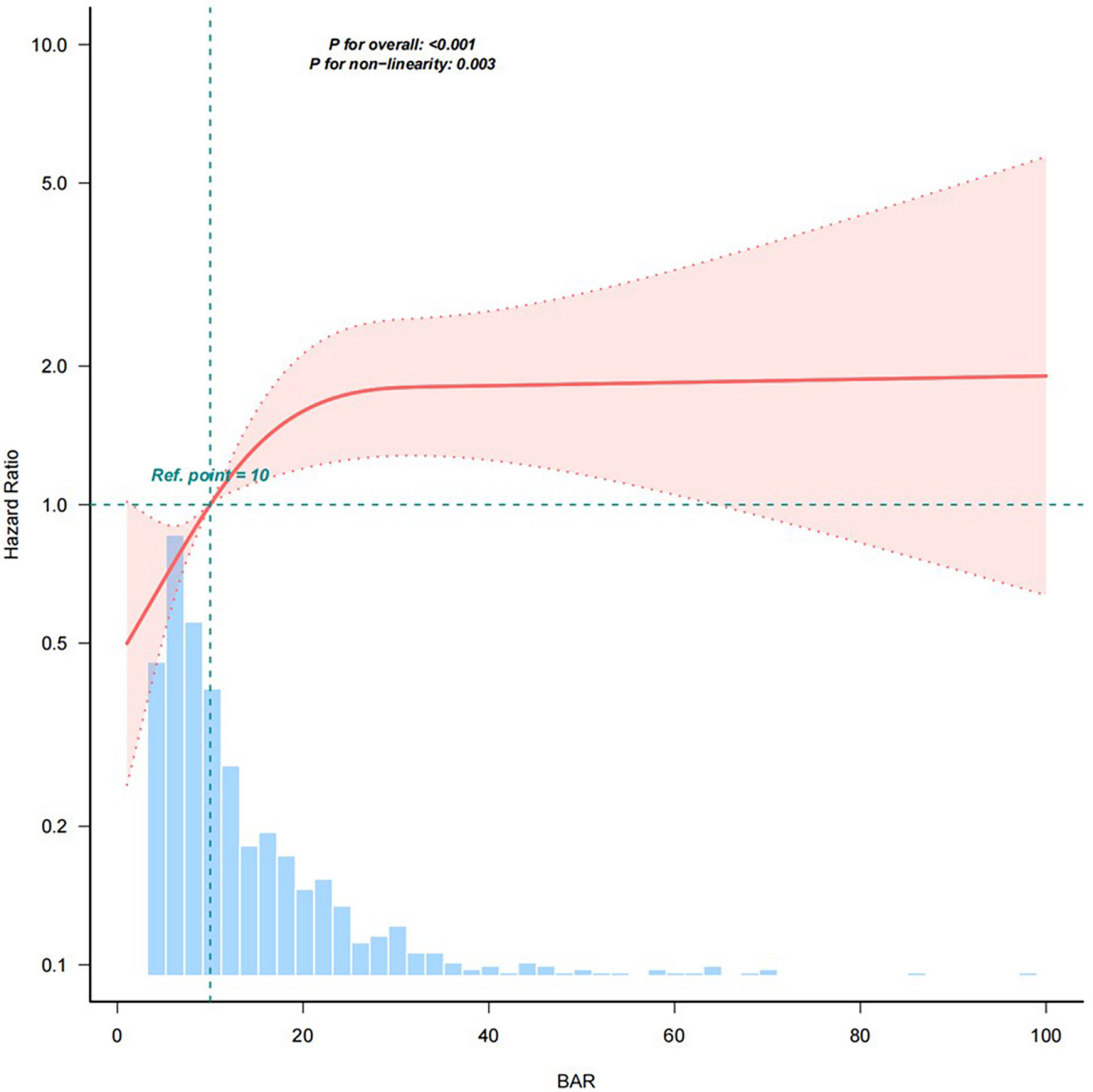
Restricted cubic spline plot of the relationship between the BAR and risk of 28-day all-cause mortality. The model was adjusted for Age, Sex, CCI, Myocardial infarct, Congestive heart failure, Peripheral vascular disease, Cerebrovascular disease, Dementia, chronic pulmonary disease, Rheumatic disease, Peptic ulcer disease, Diabetes, Paraplegia, Renal disease, Malignant cancer, Liver disease, Metastatic solid tumor, AIDS, WBC, Hb, PLT, ALP, ALT, AST, TBIL, Cr, Glu, Na^+^, K^+^, CI^−^, Ca^2+^, Lac, pH, PCO_2_, PO_2_/FiO_2_, BE, PT, PTT, Vasoactive drugs, RRT, Ventilation, Los ICU, HRT, MBP, RR, T, SPO_2_, SAPS II and SOFA. Solid lines represent the hazard ratio of 28-day all-cause mortality in patients with cardiac arrest; dotted lines represent the corresponding 95% confidence interval. Hazard ratio = 1 was set as the reference line (takes the upper limit of 100%). BAR, blood urea nitrogen to serum albumin ratio.

After adjusting for the covariates in Model 5, the results of restricted cubic spline analysis showed a nonlinear relationship between the BAR and 28-day all-cause mortality (Figure 2). As shown in Table 5, with BAR ≤ 17.981, the 28-day all-cause mortality risk increased by 5.7% (95% CI: 1.012–1.105, *P* < 0.05) for every 1-unit increase in the BAR. With BAR > 17.981, there was a 1.7% (95% CI: 0.994–1.041, *P* > 0.05) increase in the 28-day all-cause mortality risk for every 1-unit increase in the BAR; although there was an upward trend, the data did not reach statistical significance.

**Table 5:** Threshold effect analysis of the association between the BAR and 28-day all-cause mortality.

Item	HR (95% CI)	*P*-value
Turning point	17.981 (15.863, 20.099)	
BAR ≤ 17.981	1.057 (1.012, 1.105)	0.0126
BAR >17.981	1.017 (0.994, 1.041)	0.142
Likelihood Ratio test		0.007

Data presented as hazard ratio (HR) and 95% confidence interval (CI).

Adjusted for: Age, Sex, CCI, Myocardial infarction, Congestive heart failure, Peripheral vascular disease, Cerebrovascular disease, Dementia, Chronic pulmonary disease, Rheumatic disease, Peptic ulcer disease, Diabetes, Paraplegia, Renal disease, Malignant cancer, Liver disease, Metastatic solid tumor, AIDS, WBC, Hb, PLT, ALP, ALT, AST, TBIL, Cr, Glu, Na^+^, K^+^, CI^−^, Ca^2+^, Lac, pH, PCO_2_, PO_2_/FiO_2_, BE, PT, PTT, Vasoactive drugs, RRT, Ventilation, Los ICU, HRT, MBP, RR, T, SPO_2_, SAPSII and SOFA.

BAR, blood urea nitrogen to serum albumin ratio.

### 3.6 Kaplan–Meier survival curve analysis

**Figure 3:**
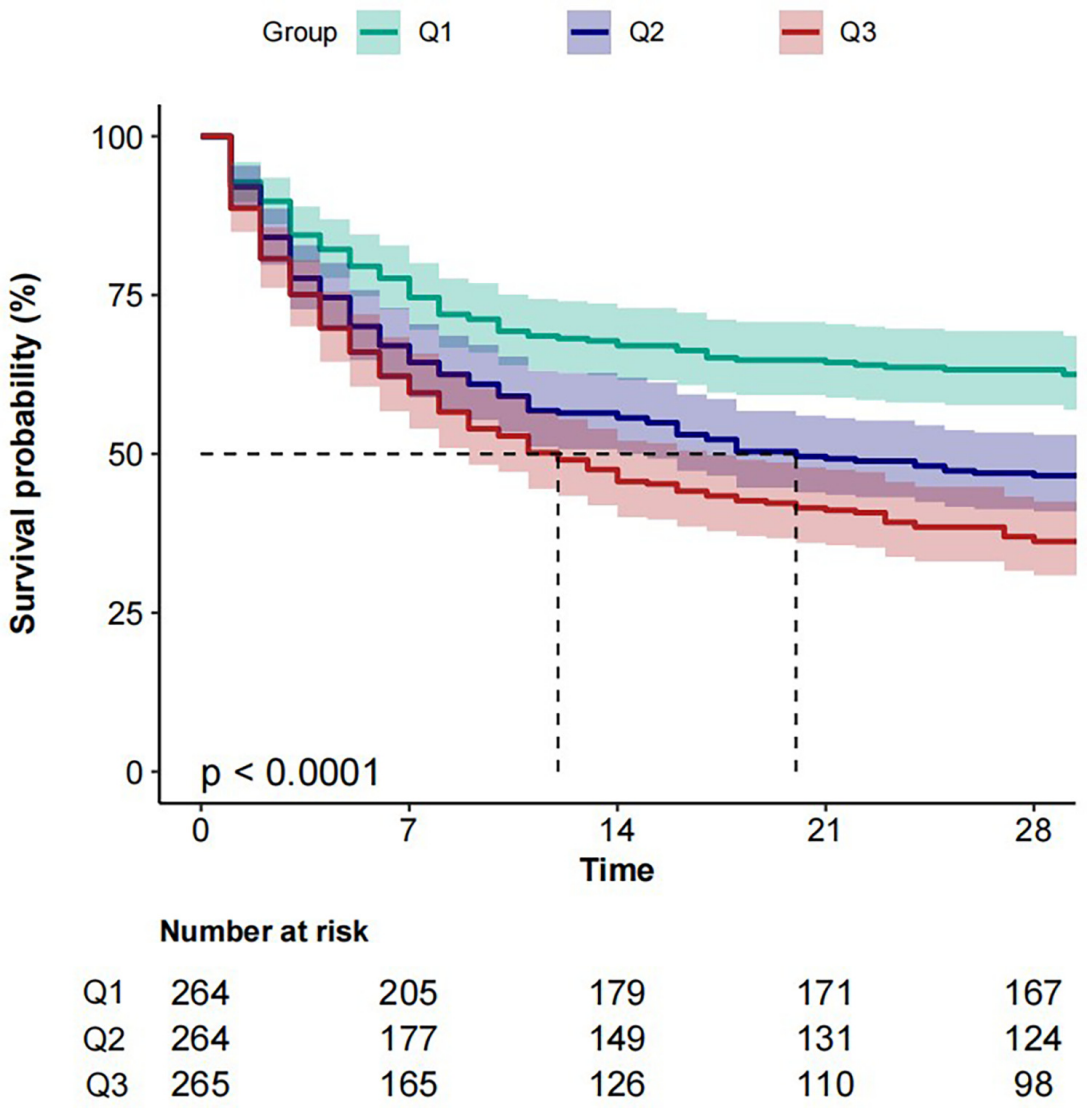
Kaplan–Meier analysis of 28-day all-cause mortality survival according to BAR group. Patients were grouped into tertiles by BAR levels (Q1, Q2, Q3). BAR, blood urea nitrogen to serum albumin ratio.

The cumulative mortality rate was compared using Kaplan–Meier curves. Following restricted cubic splines analysis, patients were divided into tertiles by BAR values (Q1, Q2, Q3). The results showed that a higher BAR (Q3) was significantly associated with an enhanced risk of 28-day all-cause mortality, compared with other BAR groups (Figure 3).

## 4 Discussion

In this retrospective cohort study, we first revealed a non-linear association between the BAR and 28-day all-cause mortality in patients with cardiac arrest. We found that BAR ≤ 17.981 was positively correlated with 28-day all-cause mortality. However, BAR > 17.981 showed no such correlation. This finding reveals a novel predictive indicator for risk stratification in patients with cardiac arrest, with important clinical implications.

BUN is the final product of protein metabolism, reflecting renal function and overall metabolic status (14), with prognostic value in heart failure surpassing that of the glomerular filtration rate and serum Cr (15, 16), and elevated levels being significantly associated with poor outcomes in cardiac arrest patients (17–20). Our analysis confirmed that BUN is an independent risk factor for 28-day mortality. As the BUN level increased, the rate of 28-day all-cause mortality showed an upward trend. ALB is the main protein in plasma, with physiological functions including anti-inflammatory and antioxidant effects, as well as the maintenance of plasma colloid osmotic pressure (21). Several studies have shown that hypoalbuminemia on admission is associated with increased in-hospital mortality (22–24), worse neurologic outcomes (24, 25), and a prolonged ICU stay (23). In this study, we also found that ALB levels in the non-survivor group were significantly lower than those in the survivor group.

Predicting the prognosis of patients with cardiac arrest using individual indicators has certain limitations. The BAR, a composite indicator composed of BUN and ALB, can comprehensively reflect the body's metabolic state, nutritional status, inflammatory response, and organ function. The BAR has been identified as an effective prognostic marker for various diseases (26–29). This study expands BAR's clinical utility by identifying its unique prognostic value in cardiac arrest patients. Cox regression analysis revealed that the BAR was positively correlated with 28-day all-cause mortality in patients with cardiac arrest, with a 1% increase in mortality risk for every 1-unit increase (HR = 1.010, 95% CI: 1.010–1.020, *P* < 0.001); this association remained consistent across multivariable-adjusted models. Kaplan–Meier curves showed that a higher BAR was significantly associated with an enhanced risk of 28-day all-cause mortality compared with other BAR groups. Huang et al. (30) observed that BAR ≥ 10.42 was significantly associated with long-term mortality in acute ischemic stroke. Wang et al. found that a BAR > 8.0 predicted 30-day and 360-day mortality in patients with sepsis (31); when BAR levels exceeded certain thresholds, mortality increased with higher BAR values. In this study, a similar trend was observed. When BAR levels decreased below 17.981, patient mortality increased; however, when BAR levels exceeded 17.981, no significant correlation was observed between the BAR and mortality.

The mechanism via which BAR increases the mortality risk in patients with cardiac arrest is not fully understood, but it may involve several potential mechanisms and related pathophysiological factors. Following cardiac arrest, the abrupt drop in cardiac output rapidly activates the renin–angiotensin–aldosterone system, sympathetic nervous system, and arginine vasopressin release (32), causing renal vasoconstriction, a reduced glomerular filtration rate (10), and enhanced sodium–water reabsorption, which markedly increases passive tubular BUN reabsorption (33). Another mechanism may involve post-resuscitation ischemia–reperfusion triggering systemic inflammation (34); cytokines disrupt the endothelium and increase permeability (34), resulting in albumin extravasation and disturbed renal hemodynamics that further elevate BUN (35). Simultaneously, the surge in reactive oxygen species induces oxidative stress, directly damaging cell membranes and proteins; additionally, reduced albumin—a key plasma antioxidant—impairs the body's defense against oxidative injury (36–38). Elevated BUN reflects renal insufficiency, metabolic disorders, and systemic inflammation, directly indicating the severity of disease. Reduced ALB reflects worsened nutritional status, uncontrolled inflammation, and impaired antioxidant defense mechanisms, directly increasing the risk of multi-organ failure. Subgroup analysis revealed an interaction between the BAR and 28-day all-cause mortality observed in RRT. This is because RRT is typically initiated in patients with severe renal dysfunction, where the clearance of BUN is compromised and the loss of ALB due to dialysis or ongoing inflammation is more pronounced.

On the basis of our threshold analysis, with BAR ≤ 17.981, each 1-unit increase was associated with a 5.7% rise in the 28-day all-cause mortality risk (HR = 1.057, *P* = 0.0126). Clinicians should implement daily BUN and albumin monitoring, optimize renal perfusion (maintaining MBP ≥ 65 mmHg), and initiate high-protein enteral nutrition within 24 h to correct hypoalbuminemia. Conversely, with BAR > 17.981 (HR = 1.017, *P* = 0.142), although further increases did not reach statistical significance, such patients remain at high overall risk and therefore require immediate multi-organ support (e.g., lung-protective mechanical ventilation, early continuous RRT), proactive complication and infection management, and multidisciplinary case reviews with structured prognostic discussions to guide precision care and timely decision-making.

Our study has several limitations. First, this study is the disproportionately high missingness rate of albumin (ALB) data, which exceeded the total enrolled cases due to inconsistent laboratory measurements across centers and retrospective data collection constraints. This degree of missingness may introduce bias despite multiple imputation, as the missing-at-random (MAR) assumption becomes less tenable when missingness exceeds 50% of observations (30, 38, 39). Consequently, conclusions derived from ALB-related analyses should be interpreted with caution. In addition, some critical variables such as the cause of cardiac arrest, response time, resuscitation time, temperature management, and body mass index (BMI) were not included in the analysis due to incomplete database records, Notably, BMI has been shown to independently predict short-term mortality in ICU populations (40). Future multicenter, large-sample, prospective studies are needed for further validation. Second, we only collected baseline indicators at admission and did not dynamically monitor the changes in BUN, ALB, and other indicators, which may affect the accuracy of prognostic assessment. Third, this study only describes the correlation between the BAR and 28-day prognosis in patients with cardiac arrest and does not establish a causal relationship.

## 5 Conclusion

In this study, we found that the relationship between the BAR and 28-day all-cause mortality in patients with cardiac arrest was non-linear. As a simple and readily available indicator, the BAR has potential clinical value in the prognostic assessment of patients with cardiac arrest.

## Data Availability

The original contributions presented in the study are included in the article/Supplementary Material, further inquiries can be directed to the corresponding author.
